# Supplementary material for: Brassicaceae transcriptomes reveal convergent evolution of super-accumulation of sinigrin
Source: Commun Biol. 2020 Dec 16;3:779. doi: 10.1038/s42003-020-01523-x (PMC7745032; doi:10.1038/s42003-020-01523-x)
Supplement: Supplementary file 1 — Supplementary Information [file 42003_2020_1523_MOESM1_ESM.pdf]

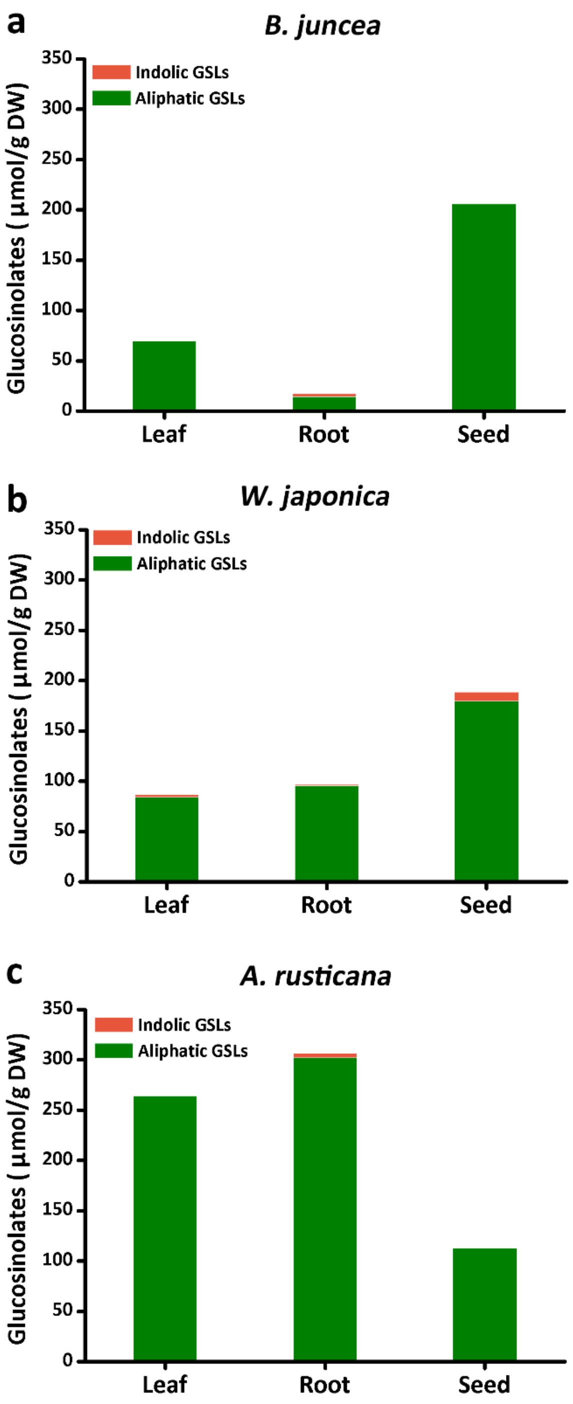

Supplementary Fig. 1. Aliphatic and indolic GSLs in leaf, root and seed of *B. juncea*, *W. japonica* and *A. rusticana*.

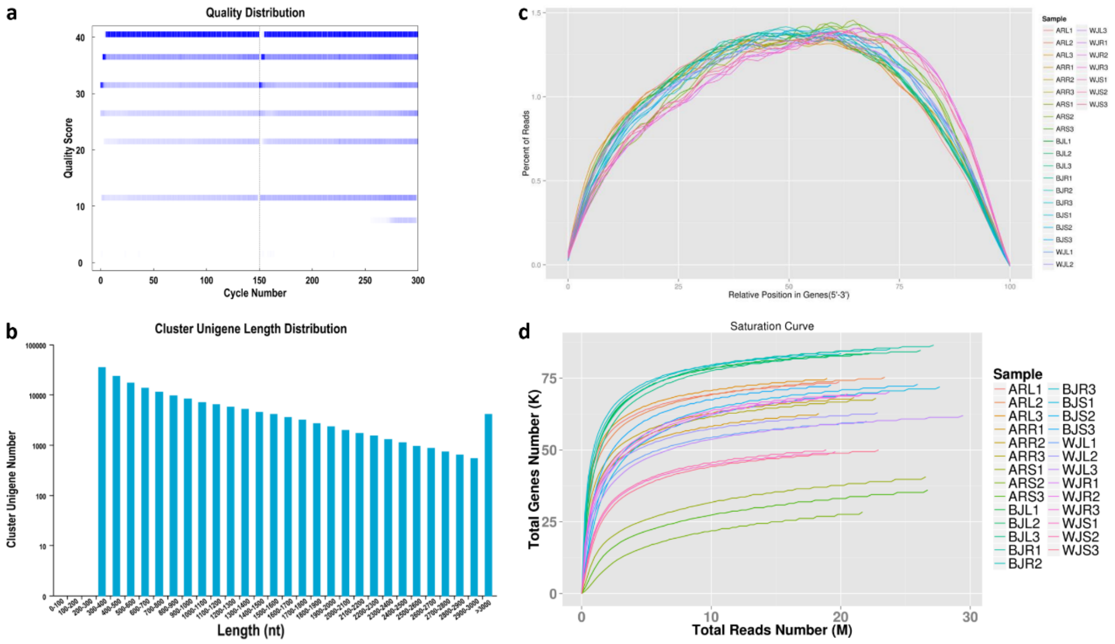

Supplementary Fig. 2. Quality assessment of RNA-seq in leaf, root and seed of *B. juncea*, *W. japonica* and *A. rusticana*. a, Quality distribution of sequencing bases. **b**, Distribution of unigenes length after transcriptome assembly. **c**, Distribution of mapped reads located in mRNAs. **d**, Simulated diagram of saturation of sequencing.

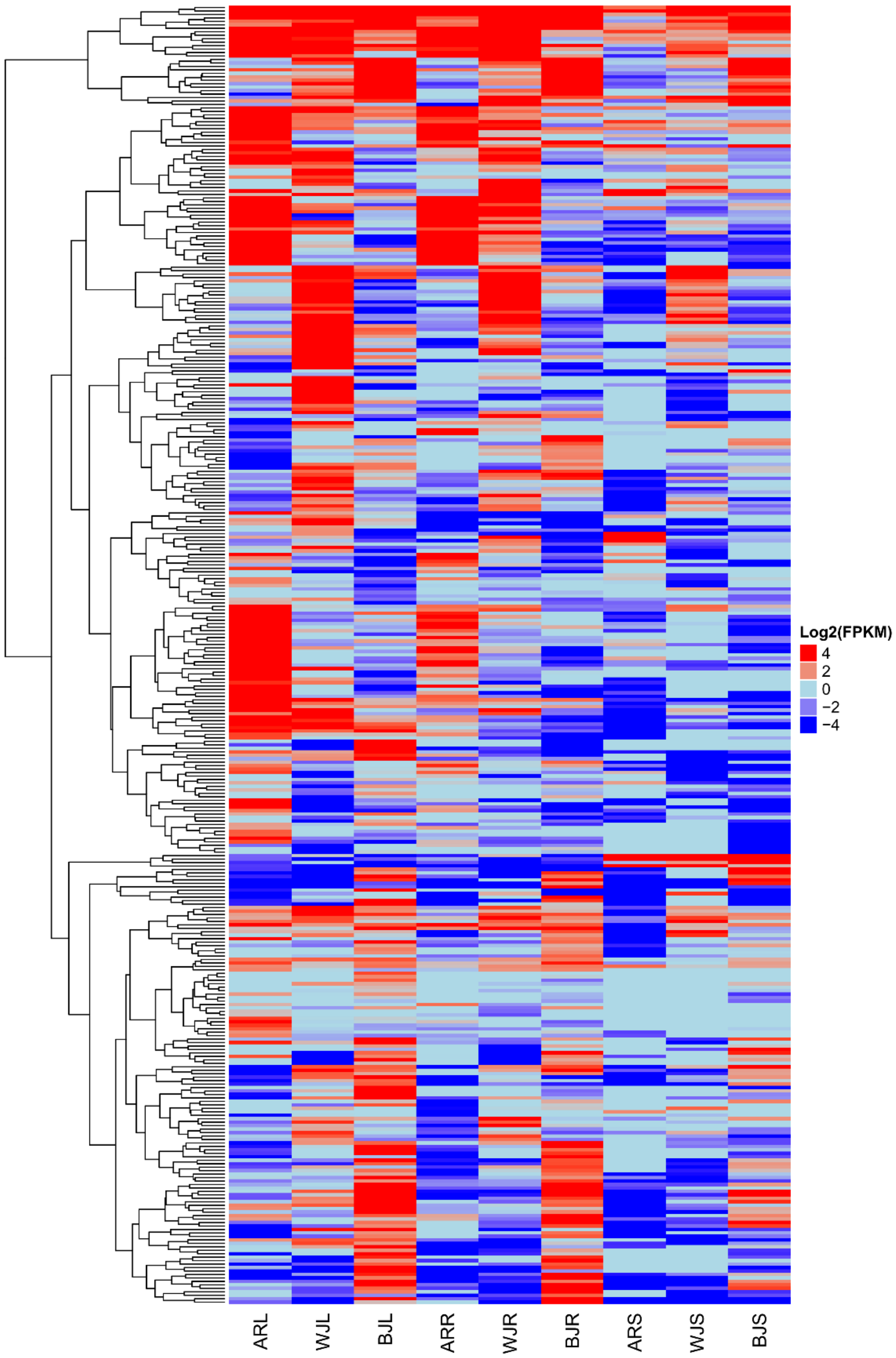

Supplementary Fig. 3. Differential expressed genes related to GSLs metabolism in *B. juncea*, *W. japonica* and *A. rusticana*. BJ, *B. juncea*. AR, *A. rusticana*. WJ, *W. japonica*. L, leaf. R, root. S, seed.

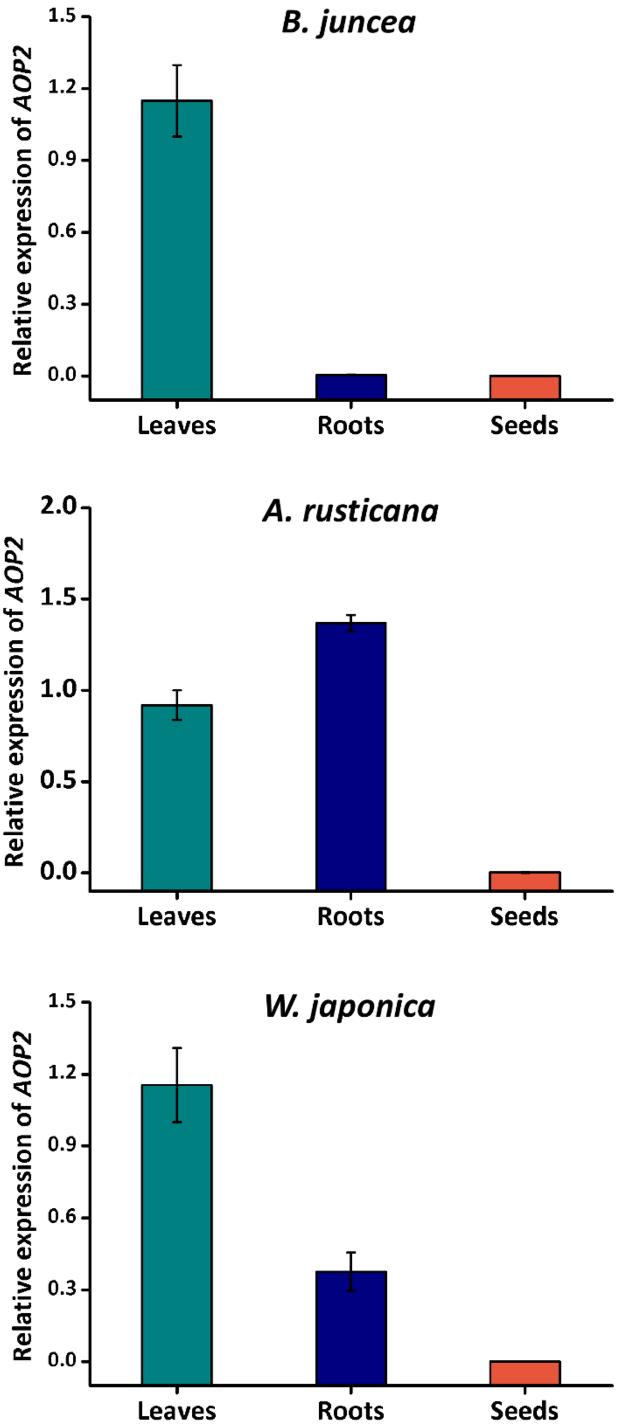

Supplementary Fig. 4. Transcriptional expression patterns of *AOP2* in leaves, roots and seeds of wasabi, horseradish and mustard species (error bars indicate standard deviation; n = 3 biologically independent samples).

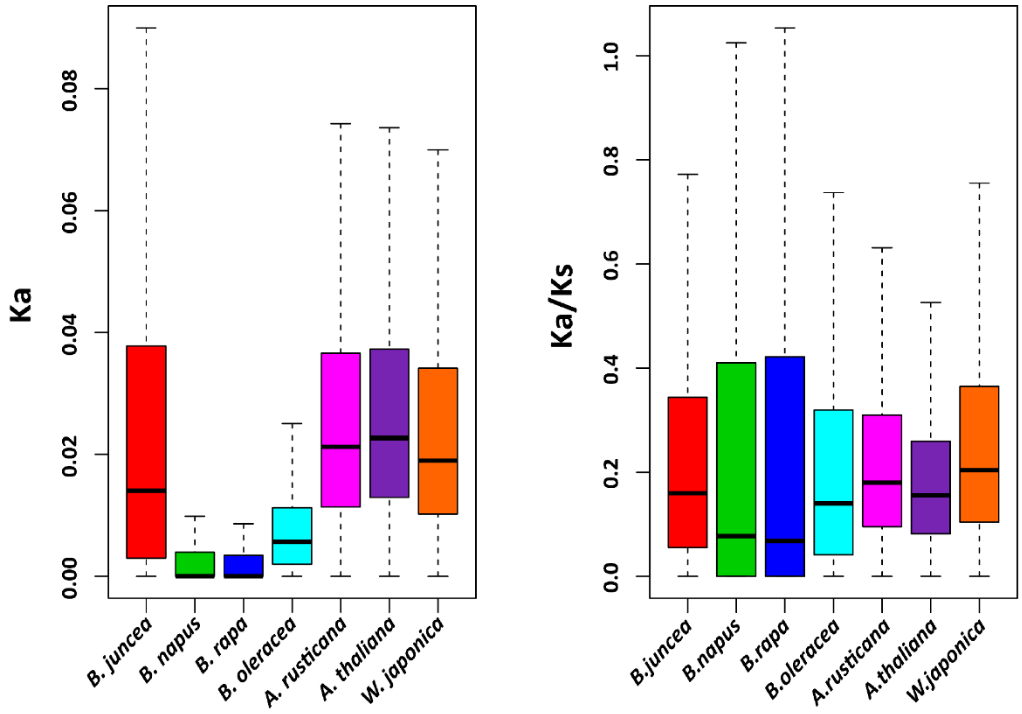

Supplementary Fig. 5. Ka and Ka/Ks calculation in seven species of *Brassicaceae* using all one-to-one orthologs.
