# Supplementary material for: Brassicaceae transcriptomes reveal convergent evolution of super-accumulation of sinigrin
Source: Commun Biol. 2020 Dec 16;3:779. doi: 10.1038/s42003-020-01523-x (PMC7745032; doi:10.1038/s42003-020-01523-x)
Supplement: Supplementary file 3 — Descriptions of Additional Supplementary Files [file 42003_2020_1523_MOESM3_ESM.pdf]

**Descriptions of Additional Supplementary Files**

**Supplementary Data 1**

**Description:** Aliphatic and indolic glucosinolate contents in the leaves, roots and seeds of *B. juncea*, *W. japonica* and *A. rusticana*.

**Supplementary Data 2**

**Description:** Glucosinolate components in the leaves, roots and seeds of *B. juncea*, *W. japonica* and *A. rusticana*.

**Supplementary Data 3**

**Description:** Statistics of sequencing reads and reads mapped to transcriptomes in all samples.

**Supplementary Data 4**

**Description:** Statistics of unigene annotation.

**Supplementary Data 5**

**Description:** Statistics of biological replicate correlations in all sequenced samples.

**Supplementary Data 6**

**Description:** Assembly estimation using 1440 BUSCO Embryophyta gene sets.

**Supplementary Data 7**

**Description:** Identification of GSL orthologs and homologs from *B. juncea*, *W. japonica* and *A. rusticana*.

**Supplementary Data 8**

**Description:** Transcriptional expression level of genes involved in glucosinolate metabolism.

**Supplementary Data 9**

**Description:** Positively selected genes (PSGs) and their annotations in *Brassicaceae*.

**Supplementary Data 10**

**Description:** Convergent genes detected by the PCOC method
